# Genome sequence of *Arthrobacter globiformis* phage MaGuCo

**DOI:** 10.1128/mra.01179-23

**Published:** 2024-02-20

**Authors:** Amanda E. Diggins, Mary G. Gubitose, Elijah G. Hinrichsen, Patrick T. Jones, Brian S. Kearns, Caitlynn E. Lord, Mary T. Parsons, Rachel A. Pitt, Isabella A. Woods, Teagan R. Zarakotas, Beth M. Wilkes

**Affiliations:** 1 Department of Natural Sciences, NHTI – Concord’s Community College, Concord, New Hampshire, USA; Loyola University Chicago, Chicago, Illinois, USA

**Keywords:** bacteriophage genetics, genome analysis, actinobacteriophage, cluster AZ

## Abstract

MaGuCo is a temperate phage isolated from soil collected in Alton, NH, USA, using *Arthrobacter globiformis*. Its genome is 43,924 base pairs long and contains 63 protein-encoding genes, 44 of which were assigned putative functions. MaCuGo is assigned to cluster AZ2 based on gene content similarity to actinobacteriophages.

## ANNOUNCEMENT

Bacteriophages (phages) are viruses that reproduce by infecting and lysing host bacterial cells (1). With increasing antibiotic resistance being found in pathogenic bacteria, the antibacterial properties of phages have garnered interest as a possible treatment alternative (2). The goal of this study was to isolate and characterize a unique phage from a local soil sample. The research was conducted primarily via protocols developed by Science Education Alliance-Phage Hunters Advancing Genomics and Evolutionary Science (3). The in-depth study of individual phage species is a crucial first step to understanding the possible medical applications of phages (2).

**Fig 1:**
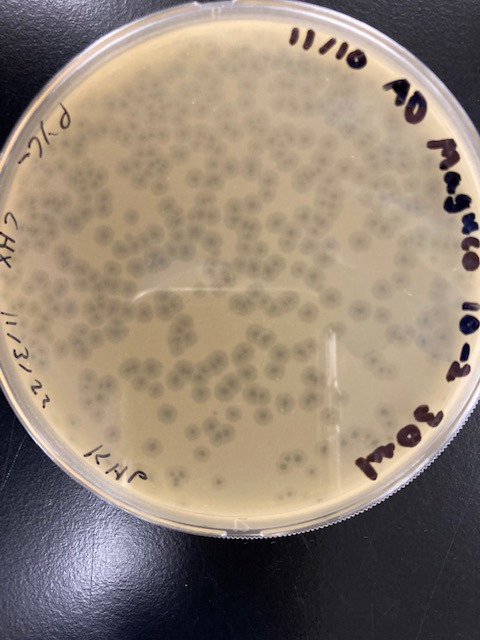
Plaque assay agar plate showing MaGuCo plaque morphology.

MaGuCo is a temperate phage from cluster AZ, subcluster AZ2 that infects *Arthrobacter globiformis* B-2979. MaGuCo was isolated in 2022 from a soil sample collected in Alton, NH, USA (43.4432 N, 71.22524 W) using standard protocols (3). Briefly, the soil sample was washed with peptone-yeast extract-calcium chloride broth, the wash was filtered through a 0.22 µm filter, and the filtrate was plated in top agar with *Arthrobacter globiformis* B-2979. MaGuCo, which results in bullseye plaques approximately 6 mm in diameter (Fig. 1) after 48 hours of incubation at 30°C, was purified through three rounds of plating. The phage was then amplified, and a high-titer lysate was collected for DNA extraction and transmission electron microscopy.

MaGuCo’s DNA was extracted from a lysate using the Phage DNA Isolation Kit from Norgen Biotek Corporation. The DNA was prepared for sequencing using the NEB Ultra II Library Kit and shotgun sequenced with an Illumina MiSeq with v3 reagents at the Pittsburgh Bacteriophage Institute to yield 353,597 150-base single-end reads that provided coverage of approximately 1,175×. Reads were assembled with Newbler v2.9 (4) and verified with Consed v29.0 (4), resulting in a genome of 43,924 base pairs (bp) long with a guanine and cytosine content of 68.2% and 3′ single-stranded overhang (5′-*CGGAGAGGCAT*-3′). Based on gene content similarity (GCS) of at least 35% to actinobacteriophages, using the GCS tool at the Actinobacteriophage database (https://phagesdb.org/), MaGuCo was assigned to cluster AZ, subcluster AZ2 (5, 6).

DNA Master v5.23.6 build 2705 (https://phagesdb.org/DNAMaster/) was used to auto-annotate the sequenced genome, and then Phage Evidence Collection and Annotation Network (PECAAN) v20221109 (7), Glimmer v3.02 (8), GeneMark v2.5p (09.08.06) (9), Starterator v1.2 (10), Phamerator v519 (11), BLASTp (using the PhagesDB and NCBI nonredundant databases) (12), HHPred (using the PDB mmCIF70, Pfam-A, and NCBI Conserved Domain databases) (13), TMHMM v2.0 (14), and TOPCONS v2.0 (15) were used to further refine the annotation and assign functions. A total of 63 genes were predicted, 44 of which were assigned a putative function. No tRNAs were found, and 19 genes were not assigned a function.

As with other cluster AZ phages, MaGuCo’s structure and assembly genes are encoded on the left third of the genome, while DNA metabolism genes are encoded on the right two-thirds, including a serine integrase. The endolysin of cluster AZ phages can be found encoded within the first third or last third of the genome. For MaGuCo, the endolysin is encoded by gene *20*. Of the 63 genes, 4 genes have no known homologs in the Actinobacteriophage and NCBI nonredundant databases (6, 12).

## Data Availability

MaGuCo is available at GenBank with accession No. OQ709203 and Sequence Read Archive (SRA) No. SRX20916068.
